# A review of the experimental evidence on the toxicokinetics of carbon monoxide: the potential role of pathophysiology among susceptible groups

**DOI:** 10.1186/s12940-018-0357-2

**Published:** 2018-02-05

**Authors:** Prabjit Barn, Luisa Giles, Marie-Eve Héroux, Tom Kosatsky

**Affiliations:** 1 National Collaborating Centre for Environmental Health, 200 - 601 West Broadway, Vancouver, BC V5Z 4C2 Canada; 2 Environmental Health Services, British Columbia Centre for Disease Control, 655 West 12th Avenue, Vancouver, BC V5Z 4R4 Canada; 3 Currently at: Department of Sport Science, Douglas College, P.O. Box 2503, 700 Royal Avenue, New Westminster, BC V3L 5B2 Canada; 4 Water and Air Quality Bureau, Health Canada, 269 Laurier Ave West, Ottawa, K1A 0K9 Canada

**Keywords:** Carbon monoxide, Carboxyhemoglobin, Indoor air guidelines, Susceptible persons, Cardiovascular disease, Chronic obstructive pulmonary disease, Anemia

## Abstract

**Background:**

Acute high level carbon monoxide (CO) exposure can cause immediate cardio-respiratory arrest in anyone, but the effects of lower level exposures in susceptible persons are less well known. The percentage of CO-bound hemoglobin in blood (carboxyhemoglobin; COHb) is a marker of exposure and potential health outcomes. Indoor air quality guidelines developed by the World Health Organization and Health Canada, among others, are set so that CO exposure does not lead to COHb levels above 2.0%, a target based on experimental evidence on toxicodynamic relationships between COHb and cardiac performance among persons with cardiovascular disease (CVD). The guidelines do not consider the role of pathophysiological influences on toxicokinetic relationships. Physiological deficits that contribute to increased CO uptake, decreased CO elimination, and increased COHb formation can alter relationships between CO exposures and resulting COHb levels, and consequently, the severity of outcomes. Following three fatalities attributed to CO in a long-term care facility (LTCF), we queried whether pathologies other than CVD could alter CO-COHb relationships. Our primary objective was to inform susceptibility-specific modeling that accounts for physiological deficits that may alter CO-COHb relationships, ultimately to better inform CO management in LTCFs.

**Methods:**

We reviewed experimental studies investigating relationships between CO, COHb, and outcomes related to health or physiological outcomes among healthy persons, persons with CVD, and six additional physiologically susceptible groups considered relevant to LTCF residents: persons with chronic obstructive pulmonary disease (COPD), anemia, cerebrovascular disease (CBD), heart failure, multiple co-morbidities, and persons of older age (≥ 60 years).

**Results:**

We identified 54 studies published since 1946. Six studies investigated toxicokinetics among healthy persons, and the remaining investigated toxicodynamics, mainly among healthy persons and persons with CVD. We identified one study each of CO dynamics in persons with COPD, anemia and persons of older age, and no studies of persons with CBD, heart failure, or multiple co-morbidities. Considerable heterogeneity existed for exposure scenarios and outcomes investigated.

**Conclusions:**

Limited experimental human evidence on the effects of physiological deficits relevant to CO kinetics exists to support indoor air CO guidelines. Both experimentation and modeling are needed to assess how physiological deficits influence the CO-COHb relationship, particularly at sub-acute exposures relevant to indoor environments. Such evidence would better inform indoor air quality guidelines and CO management in indoor settings where susceptible groups are housed.

## Background

Carbon monoxide is a colorless, odorless and tasteless gas produced from the incomplete combustion of carbon-containing fuels such as natural gas, oil, and wood. Common indoor sources include poorly maintained or improperly vented appliances such as furnaces, fireplaces, and gas stoves [1, 2]. Tobacco smoke and vehicles left idling in attached garages or near open windows and building air intakes also contribute to indoor concentrations [1, 2]. Intentional and unintentional indoor CO exposure is linked to substantial burden of disease world-wide [3–5].

Carbon monoxide toxicity is primarily mediated through hypoxic pathways, whereby CO molecules displace oxygen (O_2_) and bind to hemoglobin to form carboxyhemolgobin (COHb) [1, 6]. The concentration of COHb increases with concentration and duration of exposure. The increased presence of COHb in the blood and the resultant leftward shift in the O_2_ dissociation curve reduces O_2_ transport to and transfer at tissue sites. Other heme-containing molecules, such as myoglobin, are similarly affected [2, 7]. Organs with the highest metabolic demand, such as the heart and brain, are most sensitive to CO-induced tissue hypoxia. Less is known about non-hypoxic pathways of CO toxicity [1]. Among other direct effects, CO has been shown to activate immunological responses by interfering with nitric oxide (NO) production [8]. Clinical symptoms associated with the hypoxic effects of mild exposures are typically non-specific, and can include headache, nausea, and dizziness, while severe exposures, also called CO poisonings, can result in disorientation, unconsciousness, and cardio-respiratory arrest [1, 7]. Persons with cardiovascular (CVD) are considered most sensitive to CO exposure.

We undertook a review of indoor air guidelines for CO and their relevance to indoor settings that house susceptible persons following a CO poisoning incident that led to the evacuation and treatment of 24 occupants from one wing of a long-term care facility in Saskatchewan, Canada. Three of the evacuated residents died within a month of the incident. All three were elderly, with pre-existing co-morbidities, and CO was listed as a contributing factor in each death [9]. A power company technician was called to the facility on the morning of December 26, 2010, after several occupants reported experiencing headaches, dizziness and nausea overnight and throughout the previous day [10]. The technician measured CO concentrations of 63 ppm in the affected wing, triggering an evacuation. An investigation of the facility revealed the source to be a faulty boiler that had emitted CO into the ventilation system, which was then distributed to occupied spaces in the affected wing [10]. Carbon monoxide detectors were not required in the facility at the time of the incident and there were no specific provincial or municipal regulations requiring their use. Based on the data collected by the power company technician, which may underestimate true exposures since the facility was ventilated prior to their collection, we assume that concentrations in the facility would not have triggered CO detectors, which are designed to alarm at CO levels considered to be immediately hazardous to health [11]. This incident shows the serious consequences of indoor CO exposure among susceptible persons, even at levels below those that may be considered immediately hazardous.

The World Health Organization (WHO) recommends that indoor air not exceed CO concentrations of 86 ppm, 30 ppm, 9 ppm, and 6 ppm for exposure periods of 15-min, 1-h, 8-h and 24-h, respectively. These guidelines are intended for use in settings such as homes, offices, schools, public buildings, and health care facilities [1]. Health Canada recommends that CO levels of 25 ppm and 10 ppm not be exceeded over periods of 1-h and 24-h, respectively, in guidelines intended for residential settings [12]. Similar methodologies were used to set WHO and Health Canada guidelines. Both guidelines are set so that short and long term exposures do not lead to COHb levels above 2.0%. Derived from controlled human exposure studies, this maximum acceptable level is based on observed changes in exercise electrocardiograms in subjects with ischemic heart disease. Both sets of guidelines also used the Coburn-Forster-Kane (CFK) equation to model CO-COHb relationships. This non-linear equation uses physiological parameters that influence CO uptake and elimination and COHb formation, such as the diffusing capacity of the lungs, alveolar ventilation rate, blood volume, and partial pressure of O_2_ in the pulmonary capillaries [1, 12, 13]. Health Canada’s guidelines were based on an adapted CFK model that includes additional parameters to account for CO in alveoli and CO-bound heme proteins in extravascular spaces [14]. All modeling scenarios were based on physiological parameters relevant to healthy adults, and as such, the setting of both WHO and Health Canada guidelines did not consider the role of physiological deficits on CO-COHb relationships.

We hypothesized that six groups in particular may be at increased risk of adverse effects: persons with COPD, anemia, cerebrovascular disease (CBD), heart failure, multiple co-morbidities, and persons of older age (≥ 60 years). COPD is characterized by lower diffusion capacity, greater alveolar dead space, and a higher degree of air trapping, all of which can decrease CO elimination [1, 6]. Additionally, persons with COPD may have secondary polycythemia [15], which can increase endogenous CO production. Persons with anemia have lower hemoglobin concentrations, which leads to less O_2_ binding to hemoglobin and therefore, lower blood O_2_ content. Additionally, persons with hemolytic anemia have higher baseline COHb levels due to higher rates of endogenous CO production that result from increased heme catabolism [1, 6, 16], and persons with sickle cell anemia may have increased alveolar dead space due to impaired pulmonary capillary perfusion [17]; both conditions would increase sensitivity to CO. Finally, decreased diffusing capacity, arterial degeneration and other circulatory changes among persons with heart failure, cerebrovascular (CBV) disease, persons of older age, and/or multiple co-morbidities can lead to lower baseline O_2_ content, and in some cases, a reduced capacity to vasodilate in response to increasing CO exposures [1, 6, 18–20]. The objective of this review was to investigate evidence on the kinetics of COHb formation as a joint function of CO exposure and altered uptake, distribution, and elimination of CO and O_2_ in these groups, as well as in healthy persons and persons with CVD.

## Methods

We reviewed experimental studies in humans investigating relationships among CO, COHb, and outcomes in healthy persons, persons with CVD, and six additional susceptible groups: persons with COPD, anemia, CBD, heart failure, multiple co-morbidities, and persons of older age (≥ 60 years). Our intention was to capture all potential susceptibilities relevant to persons residing in long term care facilities. We selected susceptible groups based on (i) initial review of the rationale for indoor air quality guidelines for CO developed by the World Health Organization [1] and Health Canada [12], and ambient air quality criteria for CO developed by the U.S. Environmental Protection Agency [6], and (ii) expert consultation [21]. Our main objective was to review studies investigating CO-COHb relationships, but to capture all relevant information, we also reviewed studies investigating CO-COHb-outcome or COHb-outcome relationships. An outcome was considered to be any measured variable that could act as a surrogate for CO-related morbidity or mortality, including exercise duration, increases to cardiac output or heart rate, and cognitive effects. We identified articles though EBSCOhost (to access MEDLINE, CINAHL, PsycINFO, Biomedical Reference Collection, and Academic Search Complete), Ovid (to access Elsevier Science Direct, Evidence Based Medicine, SAGE journals online, and Cochrane Database of Systematic Reviews), and Google Scholar (to access books, book chapters, older articles, and articles from journals not indexed through major database platforms). We used broad keywords: “carboxyhemoglobin” or “carboxyhaemoglobin” and “carbon monoxide”.

The search was restricted to English language articles using an experimental study design. No date restrictions were imposed. We reviewed abstracts of studies to exclude those involving the investigation of smoking- or occupationally-related CO exposures. Additional research studies were identified by reviewing article bibliographies. The search was completed in March 2017.

## Results

**Fig. 1:**
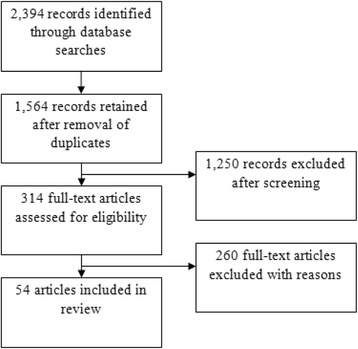
Summary of review process

Our search identified 2394 articles, of which 54 were retained, after the removal of duplicates, implementation of inclusion/exclusion criteria, and manual review of abstracts (Fig. 1). All of the studies were published between 1946 and 2016. Only ten studies were published since 2000, with the majority (27) having been published between 1970 and 1990. Most studies involved healthy adults, predominantly young males (31) [22–50], and persons with cardiovascular disease (20) [51–70]. One study was conducted among persons with COPD [71], one was conducted among persons with disease-related anemia [72], and one among healthy older persons (aged ≥60 years) [73]. We found no studies conducted among persons with CBD, heart failure, or multiple co-morbidities.

Six studies investigated CO-COHb relationships [23, 27, 35, 36, 41, 42]. All of the studies were controlled exposure studies where subjects were exposed to a known CO concentration over a given time period, and the resulting COHb levels were measured during and/or after the exposure period. Exposures varied from 12 ppm for 48-h to 6683 ppm for 3–7 min. All studies but one included only healthy males ranging from 20 to 42 years [23, 27, 36, 41, 42]. Hauck and Neuberger [35] included a 10-year old female subject, in addition to three male subjects aged 22–36 years. Individual clinical and exercise-related factors that might have impacted COHb formation were not assessed or received little attention in all of these studies. In the earliest study, Forbes et al. [23] reported that CO uptake increased with physical work and duration of exposure. The remaining five studies evaluated the CFK equation against measured COHb levels [27, 35, 36, 41, 42].

Forty-nine studies investigated CO dynamics to understand the relationships between COHb concentrations and specific outcomes. One study ([36]) investigated both CO-COHb and COHb-outcome relationships. All but two studies used a double-blind crossover design where subjects were exposed to filtered air to establish low baseline COHb levels, followed by exposure scenarios corresponding to those required to reach target COHb levels of interest. Scenarios typically involved high exposures over short periods (e.g 7500 ppm for 1 min) or lower exposures over longer periods (e.g. 36 ppm over 4 h). In most studies, no rationale was provided for the exposure scenario or target COHb level of interest. Additionally, the focus of the studies was on observation of the effects of the target COHb levels, and no studies reported the duration of effects. Two studies used non-controlled exposures [47, 53]. Thomassen et al. [47] exposed seven subjects sitting in a tent to CO generated from a camp stove. Continuous CO measurements were collected throughout the 2-h study period and venous blood samples were collected every 15-min. Aronow et al. [53] investigated outcomes after subjects were exposed to freeway air while sitting in a car for 2 h; mean (standard deviation) CO concentrations were 47 (8) ppm, compared with scenarios where subjects were exposed to filtered air for 2 h [53].

The exposure scenarios differed greatly among the subject groups studied. Table 1 summarizes CO in air levels and durations, and resulting mean COHb levels for studies investigating toxicokinetic and toxicodynamic relationships. Differences in study design, including the exposure scenarios investigated, small sample sizes, as well as lack of reporting of baseline COHb levels limit comparisons of findings between studies, however, some comparisons are possible. Five studies investigated CO exposures of 100 pm CO for 1-h. Three of these studies were conducted among subjects with CVD (*n* = 24, 30, 41) [59, 63, 65], one was conducted among healthy subjects (10) [31], and one among subjects with COPD (*n* = 10) [71]. The mean change in COHb from baseline to post-exposure was highest in a study conducted in subjects with COPD (2.6%), compared with healthy subjects (2.3%) and in subjects with CVD (1.5, 2.1, and 2.4% for the three studies). Additionally, two studies which were conducted among subjects with CVD (*n* = 15) [58] and anemia [72] investigated CO exposures of 50 ppm for 1-h, (n = 10). The mean change in COHb was higher in the study conducted in subjects with anemia (1.2%) compared with that conducted in subjects with CVD (0.9%).

**Table 1:** Summary of carbon monoxide (CO) exposure levels and durations and corresponding carboxyhemoglobin (COHb) levels, by health status

Exposure period	Exposure level (ppm)	Number of subjects	Mean^a^ COHb (%) among controls or during control period^b^	Mean^a^ COHb (%) among exposed persons or after exposure	Mean increase in COHb with exposure^c^ (%)
Healthy subjects					
1 min (5 exposures separated by 7 min each) [36]	7500	11	NR	NR	2.05
3–6.65 min [42]	6683 ppm	15	2.08 (SD: 0.08)	NR	–
2.5–3.5 min [25]	5000^d^	10	NR	3.95 (SD: 1.87)	–
5 min (× 5 exposures separated by 3 min each) [36]	1500	11	NR	NR	2.08
3–8 min [50]	3000^d^	12	NR	6.2 (SD: 0.3)	–
10–30 min [45]	4000	12	NR	10	–
30 min [49]	500^d^	15	1.2 (SD: 0.5)	8.5 (SD: 0.9)	7.3
30–45 min [46]	1200	10	–	10	–
1 h	100 [31]^d^	10	1.67 (SD: 0.33)	3.95 (SD: 0.49)	2.28
	500 [49]^d^	15	1.2 (SD: 0.5)	9.4 (SD: 0.6)	8.2
	500 [48]	13	1.2 (95% CI: 1.0, 1.4)	7.0 (95% CI: 6.5, 7.7)	5.8
4 h [34]	35.7	30	1.5 (SD: 0.27)	3.03 (SD: 0.71)	1.53
	74.1	30	1.3 (SD: 0.39)	5.1 (SD: 0.57)	3.80
10 days [33]	15^d^	15	0.5	2.4	1.90
	50^d^	15	0.5	7.2	6.70
Susceptible groups					
Subjects with cardiovascular disease					
3–120 s [51]	50,000	25	0.98	8.96	7.98
50–70 min [61]	117 (SD: 4.4)^d^	63	0.6 (SD: 0.02)	2.0 (SD: 0.05)	1.4
	253 (SD: 6.1)^d^	63	0.6 (SD: 0.02)	3.9 (SD: 0.08)	3.3
1 h	50 [58] ^d^	15	1.09 (SD: 0.15)	2.02 (SD: 0.16)	0.93
	100 [63]^d^	24	1.5	3	1.5
	100 [65]^d^	41c	1.82 (SD: 0.06)	3.93 (SD: 0.07)	2.11
	100 [59]^d^	30c	1.7	4.1	2.4
	159 (SD: 25) [68]^d^	33c	0.7	3.2	2.5
	200 [64]^d^	41	1.82 (SD: 0.06)	5.91 (SD: 0.07)	4.09
	292 (SD: 31) [68]^d^	33c	0.7	5.1	4.4
1.5 h	53 (SD: 6) [53]^d^	10	1.12 (SD: 1.20)	5.08 (SD: 1.19)	3.96
	100 [69]^d^	17	0.2–2.1	4.2 (SD: 0.3)	–
2 h	50 [55]^d^	10c	1.03 (0.27)	2.68 (SD: 0.15)	1.65
	50 [56]^d^	10	1.08	2.77	1.69
	100 [69]^d^	17	0.64	3.91	3.27
	142 ppm [39]	21	0.80 (SD: 0.20)	17.06 (SD: 1.38)	
4 h [54]	50^d^	10	1.3	2.9 (range: 1.3–3.8)	1.6
	100^d^	10	1.3	4.5 (range: 2.8–5.4)	3.2
Subjects with chronic obstructive pulmonary disease					
1 h [71] ^d^	100	10	1.48	4.08	2.60
Subjects with anaemia					
1 h [72]	50^d^	10	2.14 (SD: 0.55)	3.38 (SD: 0.83)	1.24
Subjects of older age (> 60 years)					
3 h [73]	50	36	1.30	2.77	1.47

^a^Additional summary statistics provided where reported, including standard deviation (SD), range, and 95% confidence intervals (CI). COHb levels reported to the number of decimal points reported in the original paper

^b^Represents COHb levels after exposure to fresh/filtered air, or pre-CO exposure; NR = not reported

^c^Calculated as difference between pre (baseline) and post exposure COHb levels where values were reported. Dashed line (−-) indicates that not enough information was available for calculation. The number of decimal points are presented to match values reported in the original paper

^d^Experimental conditions included exercise

The outcome measures studied also varied by clinical population. In healthy subjects, outcome measures included exercise performance, cardiac function (e.g. changes to cardiac output, heart rate, and electrocardiogram tests), respiratory response (e.g. lung capacity, minute ventilation), and nervous system response (e.g. cognitive function, behavioral impairments). For subjects with CVD, the most commonly assessed outcome was exercise-induced angina. Exercise performance was the only outcome investigated in subjects with COPD and anemia, and cognitive function was the only outcome investigated in older persons (Table 2). The lowest level for which any effect was observed was at 2%. At this level, a decrease in mean length of time to a threshold ischemic ST-segment change and decreased time to onset of angina was observed among persons with ischemic heart disease. The relationship between COHb levels > 2% COHb and outcomes were investigated for our additional groups of interest.

**Table 2:** Summary of lowest levels of carboxyhemoglobin (COHb) for which health outcomes have been described, by health status

Health outcome	Lowest mean COHb for measured response^a^ (%)	Associated CO exposure	Response
Healthy subjects			
Exercise-induced arrhythmias [44]	5 [44]	1000–3000 ppm for 4–6 min	No effect.
Exercise duration [31, 32, 40, 50]	3.95 (SD: 0.49) [31]	100 ppm for 1 h	Decrease in mean exercise duration from 698 to 663 s (*p* < 0.001).
Cardiovascular effects (cardiac output, heart rate, artery diameter, electrocardiogram changes) [25, 29, 33, 49, 89, 90]	2.4 [33]	15 ppm for 10 days (continuous exposure)	Electrocardiogram changes, specifically to *p*-waves, observed among three out of 15 subjects.
Respiratory effects [22, 25, 36, 45, 46, 89, 91]	3.95 (SD: 1.87) [25]	5000 ppm for 2.5–3.5 min	Decrease in mean (sd) inspiratory capacity from 3655 (415) ml to 3380 (419) ml (*p* < 0.05), and total lung capacity from 7705 (1083) ml to 7545 (993) ml (*p* < 0.02).
Cognitive function [24, 26, 28, 34, 37, 38, 43, 47]	5.1 (SD: 0.57) [34]	74.1 ppm for 4 h	Decreases in performance of visual tracking exercises among high exposure group, compared with low exposure and control groups (*p* < 0.01).
Cytokine production [48]	7 (95% CI: 6.5,7.7) [48]	500 ppm for 1 h	No effect.
Visual function [39]	17.06 (SD: 1.38) [39]	11,569 ppm for 4–5 min, followed by 141 ppm for 2 h	No effect.
Susceptible groups			
Subjects with cardiovascular disease			
Exercise-induced angina [53–55, 58–63, 66, 70]	2.0 (SD: 0.05) [61]	117 ppm for 50–70 min	Decrease in mean (sd) time to onset of angina from 501 (25) seconds to 482 (22) seconds (*p* = 0.054).
Exercise-induced arrhythmias [54, 61, 64, 65, 67–69]	5.91 (SD: 0.07) [65]	200 ppm for 1 h	Higher frequency of single premature ventricular contractions per hour during exercise among subjects (*p* = 0.03).
Other cardiovascular effects [51, 52, 61, 66, 69]	2.0 (SD: 0.05) [61]	117 ppm for 50–70 min	Decrease in mean (sd) length of time to a threshold ischemic ST-segment change from 576 (27) seconds to 510 (26) seconds (*p* < 0.0001).
Cognitive function [57]	3.90 [57]	100 ppm, duration not reported	Decrease in mean performance of visualization test (*p* < 0.001), but no effects on performance of speed, flexibility, digit symbol, time perception, or reaction time tests.
Exercise-induced claudication (impairment, pain discomfort in legs) [56]	2.77 [56]	50 ppm for 2 h	Decrease in mean (sd) exercise time until onset of intermittent claudication from 174 (49) to 144 (38) seconds.
Subjects with chronic obstructive pulmonary disease			
Exercise performance [71]	4.08 [71]	100 ppm for 1 h	Decrease in mean (sd) exercise duration from 219 (48) to 147 (28) seconds (*p* < 0.001).
Subjects with anemia			
Exercise performance [72]	3.38 (SD: 0.83) [72]	50 ppm for 1 h	Decrease in mean (sd) exercise duration from 221 (72) to 217 (73) seconds (*p* < 0.0001).
Subjects of older age (> 60 years)			
Cognitive function [73]	5.0 [73]	200 ppm for 1 h and 50 ppm for 2 h	No effect.

^a^Additional summary statistics provided where reported, including standard deviation (SD), range, and 95% confidence intervals (CI)

## Discussion

We reviewed evidence from 54 peer-reviewed experimental studies in humans investigating relationships between CO concentrations in air, COHb levels, and outcomes among healthy persons, those with CVD, and an additional six susceptible groups. Few studies have investigated the toxicokinetics of CO. Only six of the 54 studies identified in our search measured the relationship between CO exposure and COHb formation, and all six involved healthy participants. Since the objective of five of the six studies was to compare COHb levels predicted by the CFK model with measured levels following controlled exposures, few physiological parameters important to CO-COHb relationships were investigated. As for toxicodynamic studies, limited between-study comparison of CO-COHb levels is possible, taking into account pathophysiology. A higher mean increase in COHb from baseline to post-exposure was reported in a study of persons with COPD, compared with four studies of healthy persons or persons with CVD, at an equivalent CO exposure of 100 ppm for 1-h. Similarly, a higher mean increase in COHb was reported in a study of persons with anemia compared with a study of persons with CVD, at an equivalent CO exposure of 50 ppm for 1-h. Although findings from these studies cannot be directly compared for several reasons, including differing baseline COHb levels, the lack of subject-level data, and small sample sizes, the limited evidence does suggest that higher COHb levels may occur among some groups, likely due to underlying pathology, compared with healthy persons (or persons with CVD) at equivalent CO exposures. As we hypothesized, specific physiological deficits such as increased air trapping and greater alveolar dead space may increase CO absorption, decrease CO elimination, and/or increase COHb formation. Currently, WHO and Health Canada do not consider differences in toxicokinetics between susceptible groups in setting CO guidelines for indoor air, and instead assume that CO-COHb relationships estimated based on parameters reflecting those of healthy adults are representative of the entire population. These limited findings suggest that this may not be the case, and highlight the need for more evidence on CO-COHb relationships in susceptible groups.

Although experimental studies can allow for direct measure of CO-COHb relationships, there are limitations to the evidence these studies can provide. It is unethical to expose people to harmful CO levels, particularly over long periods of time. Additionally, it may not be appropriate to extrapolate findings on acute exposures to guidelines intended to protect persons from the effects of longer-term CO exposures, such as those intended for 24-h periods. An alternative to experimentation is the use of models that predict CO-COHb relationships. The use of models based on physiological deficits relevant to this relationship over a range of exposure scenarios can allow for a fuller understanding of CO-related susceptibility. Several empirical and mechanistic models have been developed to estimate the uptake and distribution of CO [74], including the widely used CFK equation [13, 75]. Other researchers have adapted the CFK equation to include additional physiological parameters in an attempt to more precisely predict CO-COHb relationships. An example is the setting Health Canada guidelines, which use an adapted CFK equation that incorporates parameters to account for CO in alveoli and CO-bound heme proteins in extravascular spaces [14]. Fewer studies, however, have attempted to model the effects of specific physiological deficits on CO-COHb relationships [76]. Benignus and Coleman 2010 [76] used a whole-body physiological model, which uses a form of the CFK equation, to simulate effects of CO on exercise duration among healthy persons and those with vascular disease at different exercise levels. Stenosis of the left heart arterial supply was introduced to simulate IHD, and stenosis of the cerebral arteries to simulate reductions in brain flow. For simulations involving IHD, the authors reported that the largest reductions in exercise duration were seen with the lowest severity of ischemia and COHb concentrations. For simulations involving reduced brain blood flow, a non-threshold effect on brain metabolism was seen for any increase in COHb levels when blood flow was reduced by more than 50%. In their Qualitative Risk and Exposure Assessment for Carbon Monoxide [77], the U.S. EPA used the CFK equation to model COHb levels in a simulated urban population with coronary heart disease. The population was assumed to be exposed to CO concentrations equivalent to the 8-h ambient CO standard of 9 ppm. The COHb levels in this population were estimated using a haemoglobin distribution relevant to the general population as well as an “anemic haemoglobin distribution” that was reflective of persons with anemia [77]. The percentage of the population with daily maximum COHb levels at or above 2% increased from 5.3% to 8.2% when the anemic haemoglobin distribution was used [77]. Modelling exercises such as these can be used to provide an understanding of how various sub-populations are affected under different CO exposure scenarios, which would not be possible with experimentation alone. Epidemiologic evidence can also inform our understanding of CO on susceptible persons, but must be interpreted with caution. Limited evidence suggests associations between 1 and 6 day ambient CO concentrations and increased emergency admissions for persons with COPD and sickle cell anemia [78–81]. However, recent studies have also suggested that low level ambient CO exposures, typically below 5 ppm, may be protective of some respiratory effects [82, 83]. More research is needed to assess how sub-acute and chronic CO exposures may affect these groups, including the mechanisms underlying these effects.

The majority of the studies (92%) we accessed attempted to characterize COHb-outcome relationships, predominantly among healthy persons. It is well characterized that the lowest COHb level at which an effect is seen is 2%. However, the degree to which severity of disease impacts this relationship has not been investigated. Additionally, equivalent sensitive outcomes, such as changes in ECG readings, have not been investigated in groups outside of those with CVD, making it difficult to compare CO sensitivity between potentially susceptible groups. The lack of data on CO effects among persons with multiple co-morbidities is also important, considering that individuals may have several conditions that potentially increase their overall susceptibility to CO. For example, persons with COPD are also likely to have CVD [84] and to a lesser degree, anemia [85]. The existing literature is also limited in other ways. While many studies reported mean COHb levels before and after CO exposures among all study subjects, few provided statistics on the distribution of COHb levels, including standard deviations around the mean, ranges, or subject-specific data. As noted earlier, study subjects consisted mainly of young healthy males, and where female and/or older subjects were included, reporting of results was insufficient to allow for examination of the role of sex or age on COHb-outcome relationships. Finally, much of the existing literature was published over 20 years ago. Improvements in measurement techniques have most likely led to more accurate CO and COHb measurements over time, and values reported in older studies are likely less reliable.

This review was conducted following a CO incident that was associated with three deaths in a long term care facility. Provincial or municipal regulations requiring CO detectors in Canadian health care facilities did not exist at the time of the incident. CO detector use has been shown to reduce CO poisonings in homes [86–88], and extending their use to other indoor settings would be beneficial, particularly where susceptible occupants are housed. However, CO detectors are only intended to prevent acute exposures at levels judged immediately hazardous to health, and therefore, it would be advantageous to consider ways to prevent low level exposures through comprehensive CO management programs based on education, prevention, and monitoring. In Canada as elsewhere, certified CO detectors are approved to alarm at levels considerably higher than those recommended by indoor air guidelines. Alarms are triggered when a peak value of 400 ppm is reached within 4–15 min, 150 ppm is reached within 15–50 min and when approximately 70 ppm is reached within 1–4 h [11]. These values are considered equivalent to an attained 10% COHb level, which is considerably higher than the 2% level used to derive guideline values. Certification and use of more sensitive detectors that both display and alarm at CO concentrations in the lower range would allow for better protection of the health of susceptible occupants.

## Conclusions

Our review of the experimental literature found that few studies have examined CO-COHb relationships, and in general, persons with COPD, anemia, CBD, heart failure, persons of older age, as well as those with multiple co-morbidities have largely been overlooked in the experimental CO research. Indoor air guidelines, which are largely based on limited experimental research on CO toxicodynamics, do not take into account the potential impacts of physiological deficits that alter CO uptake and elimination as well as COHb formation. Susceptibility-specific toxicokinetic modelling is needed to better understand how CO-COHb relationships differ among susceptible groups. This information in turn, can better inform indoor air guidelines as well as CO management practices in indoor settings where susceptible persons are housed.

## Abbreviations

CBD: Cerebrovascular disease
CFK: Coburn-Forster-Kane
CO: Carbon monoxide
COHb: Carboxyhemologbin
COPD: Chronic obstructive pulmonary disease
CVD: Cardiovascular disease
IHD: Ischemic heart disease
O_2_: Oxygen
WHO: World Health Organization
